# Supplementary material for: Heparin-based hydrogel scaffolding alters the transcriptomic profile and increases the chemoresistance of MDA-MB-231 triple-negative breast cancer cells
Source: Biomater Sci. 2020 Feb 13;8(10):2786–96. doi: 10.1039/c9bm01481k (PMC7497406; doi:10.1039/c9bm01481k)
Supplement: Supplementary file 2 [file BM-008-C9BM01481K-s002.zip › Supplementary File 4/HP-BhydrogelvControl/Pathways/my_analysis.Gsea.1545201079907/HALLMARK_ANDROGEN_RESPONSE.html]

Details for gene set HALLMARK\_ANDROGEN\_RESPONSE[GSEA]

|  || Dataset | expr.class.cls#HYD\_versus\_CONTROL.class.cls#HYD\_versus\_CONTROL\_repos |
| Phenotype | class.cls#HYD\_versus\_CONTROL\_repos |
| Upregulated in class | CONTROL |
| GeneSet | HALLMARK\_ANDROGEN\_RESPONSE |
| Enrichment Score (ES) | -0.4918419 |
| Normalized Enrichment Score (NES) | -2.1129053 |
| Nominal p-value | 0.0 |
| FDR q-value | 0.0 |
| FWER p-Value | 0.0 |
Table: GSEA Results Summary

  

Fig 1: Enrichment plot: HALLMARK\_ANDROGEN\_RESPONSE      
 Profile of the Running ES Score & Positions of GeneSet Members on the Rank Ordered List

  

| PROBE | DESCRIPTION (from dataset) | GENE SYMBOL | GENE\_TITLE | RANK IN GENE LIST | RANK METRIC SCORE | RUNNING ES | CORE ENRICHMENT || 1 | HOMER2 | na |  |  | 502 | 2.341 | 0.0050 | No |
| 2 | PTK2B | na |  |  | 867 | 1.982 | 0.0125 | No |
| 3 | AKT1 | na |  |  | 1297 | 1.691 | 0.0126 | No |
| 4 | PTPN21 | na |  |  | 1479 | 1.590 | 0.0244 | No |
| 5 | RRP12 | na |  |  | 2114 | 1.308 | 0.0087 | No |
| 6 | SRF | na |  |  | 3494 | 0.920 | -0.0511 | No |
| 7 | PMEPA1 | na |  |  | 4320 | 0.748 | -0.0843 | No |
| 8 | UBE2I | na |  |  | 4367 | 0.738 | -0.0768 | No |
| 9 | ACTN1 | na |  |  | 4559 | 0.699 | -0.0775 | No |
| 10 | ZBTB10 | na |  |  | 4606 | 0.692 | -0.0706 | No |
| 11 | H1F0 | na |  |  | 5247 | 0.593 | -0.0962 | No |
| 12 | ADRM1 | na |  |  | 5890 | 0.501 | -0.1231 | No |
| 13 | CDC14B | na |  |  | 5959 | 0.492 | -0.1201 | No |
| 14 | NKX3-1 | na |  |  | 6599 | 0.403 | -0.1481 | No |
| 15 | HERC3 | na |  |  | 6643 | 0.402 | -0.1450 | No |
| 16 | ABCC4 | na |  |  | 7351 | 0.319 | -0.1778 | No |
| 17 | CCND1 | na |  |  | 7494 | 0.308 | -0.1811 | No |
| 18 | ZMIZ1 | na |  |  | 7808 | 0.271 | -0.1938 | No |
| 19 | CAMKK2 | na |  |  | 7928 | 0.258 | -0.1966 | No |
| 20 | IQGAP2 | na |  |  | 7960 | 0.254 | -0.1948 | No |
| 21 | STEAP4 | na |  |  | 8443 | 0.206 | -0.2173 | No |
| 22 | MAP7 | na |  |  | 8695 | 0.182 | -0.2280 | No |
| 23 | ADAMTS1 | na |  |  | 8727 | 0.178 | -0.2272 | No |
| 24 | NGLY1 | na |  |  | 9082 | 0.143 | -0.2439 | No |
| 25 | SGK1 | na |  |  | 9100 | 0.141 | -0.2429 | No |
| 26 | ANKH | na |  |  | 9443 | 0.098 | -0.2594 | No |
| 27 | ALDH1A3 | na |  |  | 9613 | 0.087 | -0.2671 | No |
| 28 | CENPN | na |  |  | 9626 | 0.085 | -0.2666 | No |
| 29 | FKBP5 | na |  |  | 9936 | 0.048 | -0.2821 | No |
| 30 | MAK | na |  |  | 9959 | 0.045 | -0.2827 | No |
| 31 | RAB4A | na |  |  | 10685 | -0.028 | -0.3203 | No |
| 32 | CDK6 | na |  |  | 10768 | -0.034 | -0.3241 | No |
| 33 | INSIG1 | na |  |  | 11030 | -0.071 | -0.3368 | No |
| 34 | FADS1 | na |  |  | 11338 | -0.094 | -0.3516 | No |
| 35 | UAP1 | na |  |  | 11350 | -0.096 | -0.3509 | No |
| 36 | CCND3 | na |  |  | 11490 | -0.109 | -0.3567 | No |
| 37 | DHCR24 | na |  |  | 11725 | -0.142 | -0.3671 | No |
| 38 | PA2G4 | na |  |  | 11816 | -0.152 | -0.3698 | No |
| 39 | SPDEF | na |  |  | 11987 | -0.171 | -0.3764 | No |
| 40 | HMGCS1 | na |  |  | 12129 | -0.190 | -0.3812 | No |
| 41 | MERTK | na |  |  | 12246 | -0.205 | -0.3845 | No |
| 42 | KRT8 | na |  |  | 12716 | -0.245 | -0.4058 | No |
| 43 | AKAP12 | na |  |  | 13048 | -0.290 | -0.4193 | No |
| 44 | TMPRSS2 | na |  |  | 13211 | -0.312 | -0.4236 | No |
| 45 | INPP4B | na |  |  | 13343 | -0.328 | -0.4260 | No |
| 46 | B4GALT1 | na |  |  | 13415 | -0.340 | -0.4252 | No |
| 47 | SAT1 | na |  |  | 13558 | -0.362 | -0.4278 | No |
| 48 | HMGCR | na |  |  | 13559 | -0.362 | -0.4230 | No |
| 49 | STK39 | na |  |  | 13622 | -0.374 | -0.4212 | No |
| 50 | ABHD2 | na |  |  | 14131 | -0.431 | -0.4420 | No |
| 51 | SEC24D | na |  |  | 14308 | -0.465 | -0.4450 | No |
| 52 | TSC22D1 | na |  |  | 14324 | -0.468 | -0.4396 | No |
| 53 | SRP19 | na |  |  | 14434 | -0.480 | -0.4388 | No |
| 54 | ARID5B | na |  |  | 14668 | -0.519 | -0.4441 | No |
| 55 | SCD | na |  |  | 15219 | -0.600 | -0.4649 | No |
| 56 | GSR | na |  |  | 15538 | -0.659 | -0.4727 | No |
| 57 | SMS | na |  |  | 15905 | -0.723 | -0.4822 | Yes |
| 58 | XRCC6 | na |  |  | 15966 | -0.737 | -0.4755 | Yes |
| 59 | PIAS1 | na |  |  | 16126 | -0.773 | -0.4734 | Yes |
| 60 | IDI1 | na |  |  | 16426 | -0.838 | -0.4779 | Yes |
| 61 | VAPA | na |  |  | 16607 | -0.874 | -0.4756 | Yes |
| 62 | APPBP2 | na |  |  | 16847 | -0.940 | -0.4756 | Yes |
| 63 | KRT19 | na |  |  | 16930 | -0.965 | -0.4670 | Yes |
| 64 | ITGAV | na |  |  | 16980 | -0.980 | -0.4564 | Yes |
| 65 | ELK4 | na |  |  | 17009 | -0.987 | -0.4447 | Yes |
| 66 | ACSL3 | na |  |  | 17071 | -1.007 | -0.4344 | Yes |
| 67 | SLC26A2 | na |  |  | 17094 | -1.013 | -0.4220 | Yes |
| 68 | TNFAIP8 | na |  |  | 17185 | -1.045 | -0.4127 | Yes |
| 69 | DNAJB9 | na |  |  | 17375 | -1.111 | -0.4078 | Yes |
| 70 | SLC38A2 | na |  |  | 17461 | -1.138 | -0.3970 | Yes |
| 71 | PDLIM5 | na |  |  | 17595 | -1.194 | -0.3880 | Yes |
| 72 | NDRG1 | na |  |  | 17623 | -1.208 | -0.3733 | Yes |
| 73 | BMPR1B | na |  |  | 17678 | -1.234 | -0.3596 | Yes |
| 74 | TPD52 | na |  |  | 17682 | -1.234 | -0.3433 | Yes |
| 75 | ELL2 | na |  |  | 17763 | -1.268 | -0.3305 | Yes |
| 76 | SPCS3 | na |  |  | 17860 | -1.315 | -0.3179 | Yes |
| 77 | GNAI3 | na |  |  | 17910 | -1.337 | -0.3026 | Yes |
| 78 | UBE2J1 | na |  |  | 17956 | -1.352 | -0.2869 | Yes |
| 79 | NCOA4 | na |  |  | 18359 | -1.611 | -0.2864 | Yes |
| 80 | RPS6KA3 | na |  |  | 18422 | -1.654 | -0.2675 | Yes |
| 81 | MYL12A | na |  |  | 18440 | -1.665 | -0.2462 | Yes |
| 82 | ELOVL5 | na |  |  | 18504 | -1.707 | -0.2266 | Yes |
| 83 | LMAN1 | na |  |  | 18569 | -1.757 | -0.2065 | Yes |
| 84 | XRCC5 | na |  |  | 18640 | -1.834 | -0.1856 | Yes |
| 85 | PGM3 | na |  |  | 18780 | -1.983 | -0.1664 | Yes |
| 86 | LIFR | na |  |  | 18803 | -2.023 | -0.1405 | Yes |
| 87 | TMEM50A | na |  |  | 18905 | -2.174 | -0.1167 | Yes |
| 88 | GPD1L | na |  |  | 18914 | -2.191 | -0.0878 | Yes |
| 89 | SORD | na |  |  | 18930 | -2.228 | -0.0588 | Yes |
| 90 | B2M | na |  |  | 19069 | -2.604 | -0.0312 | Yes |
| 91 | DBI | na |  |  | 19117 | -2.835 | 0.0042 | Yes |
Table: GSEA details [plain text format]

  

Fig 2: HALLMARK\_ANDROGEN\_RESPONSE      
 Blue-Pink O' Gram in the Space of the Analyzed GeneSet

  

Fig 3: HALLMARK\_ANDROGEN\_RESPONSE: Random ES distribution      
 Gene set null distribution of ES for **HALLMARK\_ANDROGEN\_RESPONSE**
